# Exploration of Metal-Doped Iron Oxide Nanoparticles as an Antimicrobial Agent: A Comprehensive Review

**DOI:** 10.7759/cureus.69556

**Published:** 2024-09-16

**Authors:** Iram Saba, Khalid M Batoo, Kaiser Wani, Ritesh Verma, Saif Hameed

**Affiliations:** 1 Biotechnology, Amity Institute of Biotechnology, Amity University Haryana, Gurugram (Manesar), IND; 2 Research and Scientific Center, Sultan Bin Abdulaziz Humanitarian City, Riyadh, SAU; 3 Medical Physics, King Abdullah Institute for Nanotechnology, King Saud University, Riyadh, SAU; 4 Biotechnology, University Institute of Biotechnology, Chandigarh University, Mohali, IND; 5 Biochemistry, College of Science, King Saud University, Riyadh, SAU; 6 Physics, Amity University Haryana, Gurugram (Manesar), IND

**Keywords:** antibacterial activity, iron oxide, nanoparticles, reactive oxygen species, toxicity

## Abstract

Over the past two decades, nanotechnology has captured significant interest, especially in the medical field, where the unique characteristics of nanoscale particles offer substantial advantages. The family of nanosized materials, specifically iron oxide nanoparticles (IONPs), has emerged as promising due to their magnetic properties, biocompatibility, and substantial surface area for therapeutic molecule attachment. The review explores various strategies to enhance the antibacterial properties of IONPs, such as metal doping, which modifies their physicochemical, biological, electrical, and optical properties. Metal-doped IONPs, including those with nickel, copper, zinc, selenium, molybdenum, gold, and others, have shown that they effectively eradicate viruses and bacteria. The mechanisms behind their enhanced antibacterial activity involve generating reactive oxygen species (ROS), inhibiting antibiotic-resistant genes, disrupting cell walls and DNA, dysfunction of efflux pumps, and internalizing nanoparticles. The review also addresses the potential toxicity of IONPs, highlighting factors such as their dimension, form, and outermost layers, which change how they affect the overall condition of cellular structures. Surface coatings using polymers and essential oils are among the strategies being investigated as potential ways to reduce toxicity.

This review additionally looks into IONPs' drug delivery potential for antibiotics and antifungals. The integration of IONPs with various pharmaceutical compounds and their controlled release mechanisms are also detailed. The review concludes by offering a positive outlook on the potential enhancements and prospects of IONPs. Challenges in synthesis technologies, size tuning, and surface alteration are acknowledged, emphasizing the need for continued research to fully harness the capabilities of IONPs in biomedical applications.

## Introduction and background

Researchers have sparked significant curiosity in nanotechnology in the last 20 years. It is an intriguing field of scientific study. The distinctive attributes of nanoscale particles are leading to their widespread use in many industries, most notably in the medical industry. Nanomaterial-based medicines are smaller and easier to absorb. Nanoparticles are more versatile and adaptable than bare particles, and their physicochemical properties may be modified easily [1]. Changes to nanoparticle characteristics have been extensively studied to improve their uses. Many different types of nanoparticles, each with its own set of physiochemical traits, are used in the study of drug delivery. Alternatives include metal oxide nanoparticles such as zinc oxide and titanium dioxide, carbon nanotubes, fullerenes, quantum dots, albumin linkages, and liposomes [2].

The family of nanosized materials known as iron oxide nanoparticles (IONPs) is among the most interesting. These nanoparticles hold great promise because of their magnetic characteristics, which can be carefully adjusted. In addition, IONPs demonstrate exceptional biocompatibility and offer a large surface area that theranostic molecules may cling to [3]. Gentamicin reveals how conjugating IONPs with antibiotics boosts antibacterial activity [4]. It was more bacteriostatic against Gram-positive *Bacillus subtilis* and *Staphylococcus aureus *than Gram-negative Escherichia coli. When gentamicin is conjugated, the lowest inhibitory concentration against all strains drops approximately ten times [4]. In some instances, IONP-antibiotic conjugation backfires. Amoxicillin-conjugated IONPs grew *Pseudomonas aeruginosa* and *S. aureus* [5]. Organic acids such as humic acid promote microorganisms. Overall, additives, coatings, and conjugates may significantly impact IONPs' antibacterial activity, which is promising [6]. Doping IONPs with metal atoms also improves their magnetic characteristics and antibacterial activities. Doping is the controlled injection of an outside element into a material's crystal lattice to change its characteristics [7]. Similarly, including external metal particles in IONPs modifies their physical, chemical, biological, electrical, and optical properties, resulting in enhanced performance compared to IONPs without any additions. Many metals and their oxides, including nickel (Ni) [8], copper (Cu) [9], Cobalt (Co) [10], zinc (Zn) [11], selenium (Se) [12], molybdenum (Mo) [13], gold (Au) [14], zinc oxide (ZnO) [15], and copper oxide (CuO), are doped into IONPs [16]. These compounds have been found to possess antimicrobial properties against pathogenic bacteria.

**Figure 1:**
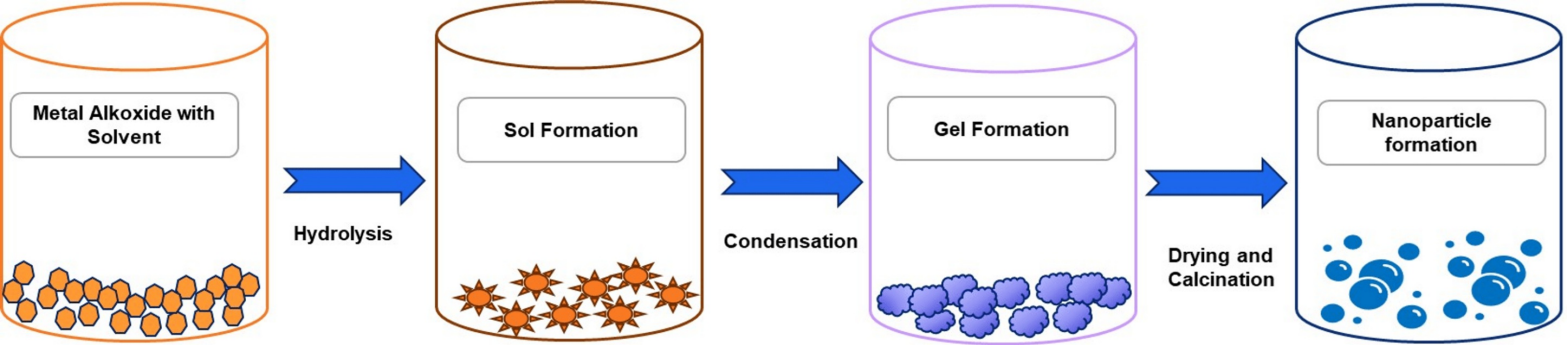
Synthesis of nanoparticles using the sol-gel method The image was created using Microsoft PowerPoint 3.12

Earlier studies indicate that metal doping can increase the production of reactive oxygen species (ROS), which may enhance various biological functions, including antibacterial and anticancer activities [8-16]. Nanoparticles can be doped using techniques such as sol-gel, laser ablation, green synthesis, and microwave-assisted synthesis. The sol-gel process is the best way to make doped nanoparticles because it is easy to use and gives you much control over the particles' size and shape [17]. The sol-gel process makes different nanomaterials, especially metal oxide nanoparticles. The molecular precursor, metal alkoxide, is mixed with water or alcohol in this method. It is then heated and stirred into a gel, as shown in Figure 1. According to studies, green nanoscale metal production is possible using plant resources. Recently, green nanoscale metal production has been intensively researched. Low yield, non-uniform particle size distribution, complicated extraction processes, seasonal and local raw material availability, and other issues must be solved for the practical production and deployment of green-synthesized nanomaterials. Thus, further research should take advantage of inexpensive raw materials, straightforward energy-saving methods, and enhanced nanoscale metal particle manufacturing [18]. From this perspective, we combed over the research on metal-doped IONPs' antibacterial properties and the mechanisms by which they may combat harmful microorganisms. We reviewed the various effects of stimuli and the probable disadvantages of using metals as doping agents. We also suggested potential areas for improvement in enhancing the biocompatibility of doped IONPs in the future.

## Review

Doped nanoparticles: the future of antibacterial agents

This review centers on the antibacterial applications of the doped nanoparticles. These particles demonstrate properties such as enhanced cellular internalization ability, lack of cytotoxicity, and improved binding capacity. Most research points to the primary cause of doped nanoparticles' increased antibacterial activity as their capacity to generate ROS. ROS are significantly involved in the immune system's protection against infections. Conversely, antioxidants have long been acknowledged for safeguarding the host organism from infections. Understanding the characteristics of the bacteria and their sensitivity to ROS could help unravel this discrepancy. While ROS successfully eliminates some bacteria, others survive under oxidative conditions. It is critical to understand how ROS eliminate bacteria and how particular microbes depend on ROS for their continued existence [19].

**Figure 2:**
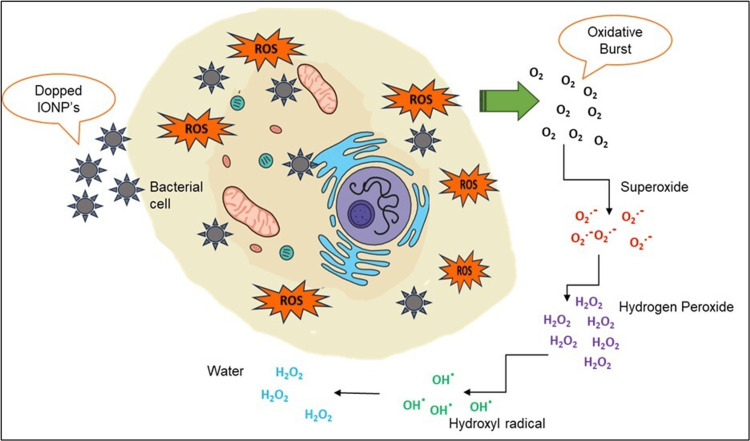
ROS generation upon doped IONPs The image was created using Microsoft PowerPoint 3.12 ROS, reactive oxygen species; IONP, iron oxide nanoparticle

Phagocytes are present in tissues or can be attracted through inflammatory mechanisms. Phagocytes identify microbes by detecting various molecular patterns exhibited by the microbes and attempting to engulf them. Upon phagocytosis of a microbe, the subsequent course of action within the phagosome is determined by the specific molecules present on the microbe's surface. NADPH oxidase, also known as NADPH oxidase 2, generates ROS upon detecting bacteria. This approach serves as a method for eradicating various forms of pathogens. For instance, dectin-1 on phagocytes' surface may unite with β-glucan found on fungi [20]. Upon phagocytosis of a pathogen, it is necessary for the pathogen to either evade the respiratory burst, endure its oxidative effects, or find a way to exit the phagosome to ensure its survival. ROS production occurs due to the immune system's hyperdrive response to pathogen detection. Multiple cellular compartments, including the mitochondria, contribute to the production of ROS and the respiratory burst of phagocytes. These ROS act as intermediates in several signal transduction pathways, including the signaling mediated by leukocyte pattern recognition receptors. Neutrophil extracellular traps cannot form without the generation of ROS [21]. The generation of ROS by introducing dopants changes the cellular redox potential, leading to variations in cell responses and, ultimately, cell apoptosis. The variation in the synthesis method is a crucial factor, as it can potentially modify the crystal vacancies and dopant percentage. The following section will discuss some significant dopant materials, particularly doped IONP, their proposed mechanism, and synthesis methods shown in Figure 2.

Doped iron oxide nanoparticles

Doped IONPs are made up of iron oxides whose structure has been altered by adding other metals or elements. Nanoparticles may have their physicochemical, biological, electrical, and optical properties altered by adding dopants, which are foreign metals, to an otherwise empty crystal lattice [22]. Several synthesis approaches are used to produce doped IONPs: coprecipitation, green combustion, sol-gel process, chemical polymerization approach, microwave-assisted procedure, and hydrothermal approach [23]. Doped IONPs are employed in drug administration, antibacterial, anticancer, and MRI imaging. Doped IONPs are usually antimicrobial compounds made of transition metals due to their bulk antibacterial properties. This is because transition metals can destroy microorganisms [24]. Casula et al. state that naked IONPs can be doped to gain inherent magnetic features and desired morphological or structural qualities [25]. This modification could enhance the antibacterial activity of doped IONPs. In addition, Ahghari et al. have shown that doped IONPs produce a higher yield compared to naked IONPs [26]. By lowering the energy band gap and joining spins and electric charges, adding metals to nanoparticles can change their size and magnetic properties. Also, smaller metal-doped nanoparticles tend to have better antibacterial properties because they have more surface area than volume, making it easier for them to interact with bacterial cells. Doping with metal can also make magnetic nanoparticles have more charged particles [22].

Effects of doped IONPs on antimicrobial activity

The intrinsic characteristics of bare nanoparticles may be increased by doping them with foreign metals. Improving antibacterial activity using various doping techniques is one of the noticeable changes [27]. Doped IONPs 20-200 nm offers antibacterial activity against many microorganisms. Activity is higher at sizes under 20 nm. Doped IONPs greater than 200 nm have little antibacterial activity. Transition metal-doped IONPs have substantial antibacterial action. This action relies on nanoparticle manufacturing, size, concentration, and application [28-30].

Nanoparticles affect bacteria differently depending on the strain or species. The particular cell walls and membranes of pathogens make them sensitive to nanoparticles. The polarity of Gram-positive bacteria's cell wall and membrane may reduce their resistance to IONPs [31-32]. Gram-negative bacteria's cell walls are physically and chemically complex, comprising phospholipid proteins, lipopolysaccharides, and a thin peptidoglycan layer [33]. Outside cell membranes of Gram-negative bacteria contain lipopolysaccharides. Increased negative charge by lipopolysaccharides prevents free radicals from entering [34]. Because of these characteristics, IONPs are less likely to impact Gram-negative bacteria. Gram-positive bacteria are more susceptible to lower concentrations of peptidoglycan since they predominantly depend on it [35].

Metal-doped IONPs' antibacterial activity has shown encouraging outcomes against resistant microorganisms. *Bacillus subtilis*, *Klebsiella pneumonia*, *E. coli*, *S. aureus*, *Proteus vulgaris*, and other bacteria are efficiently inhibited in their development by a variety of metal-doped IONPs, including Zn, Cu, Ni, Se, Au, chromium (Cr), and calcium (Ca). This was shown by employing the zone of inhibition test. Nevertheless, the efficacy of molybdenum (Mo)- and Co-doped IONPs differed. Among all the metal-doped IONPs, Zn-doped IONPs, Cu-doped IONPs, and Se-doped IONPs have shown the most robust bacterial suppression, as demonstrated by a zone of inhibition of at least 20 nm [36-37]. Table 1 lists the several doped IONPs with antibacterial activity at different doses. Potential action mechanisms are also included.

**Table 1:** Antimicrobial activity and mechanism of some of the doped IONPs AU, gold; Cu, copper; NDG, nitrogen-doped graphene; Ni, nickel; PEI, polyethyleneimine; ROS, reactive oxygen species; ZNo, zinc oxide; ZOI, zone of inhibition; Fe_3_O_4_, ferrous ferric oxide; Fe_2_O_3_, ferric oxide

Doped IONPs						
Zn-doped						
Nanoparticle	Coating material	Organism	Concentration Of nanoparticles (µg)	ZOI (mm)	Mechanisms	Study
ZnO-doped Fe_3_O_4_	Ag	E. coli	250,000	19	Nanocomposites made of ZnO stick together at the cell wall or cytoplasm of bacteria. This leads to the release of Zn2+ ions, which help bacteria die and damage membranes.	[32]
		S. aureus	250,000	11		
Cu-doped						
Cu-doped α- Fe_2_O_3_	PEI	P. vulgaris	400	16	The particles damage the cell wall, which leads to reactive stress and cell death. It makes ROS, which helps proteins and DNA oxidase.	[33]
		Serratia marcescens	400	16		
		Streptococcus mutants	400	10		
Ni-doped						
Ni-doped Fe_3_O_4_	NDG, cellulose	E. coli	50	16	Ni2+ and Fe3+ ions interact with membrane proteins and break down the bacterial membrane. This makes it harder for bacteria to grow.	[5]
		B. subtilis	50	14		

The antimicrobial action of doped IONPs

**Figure 3:**
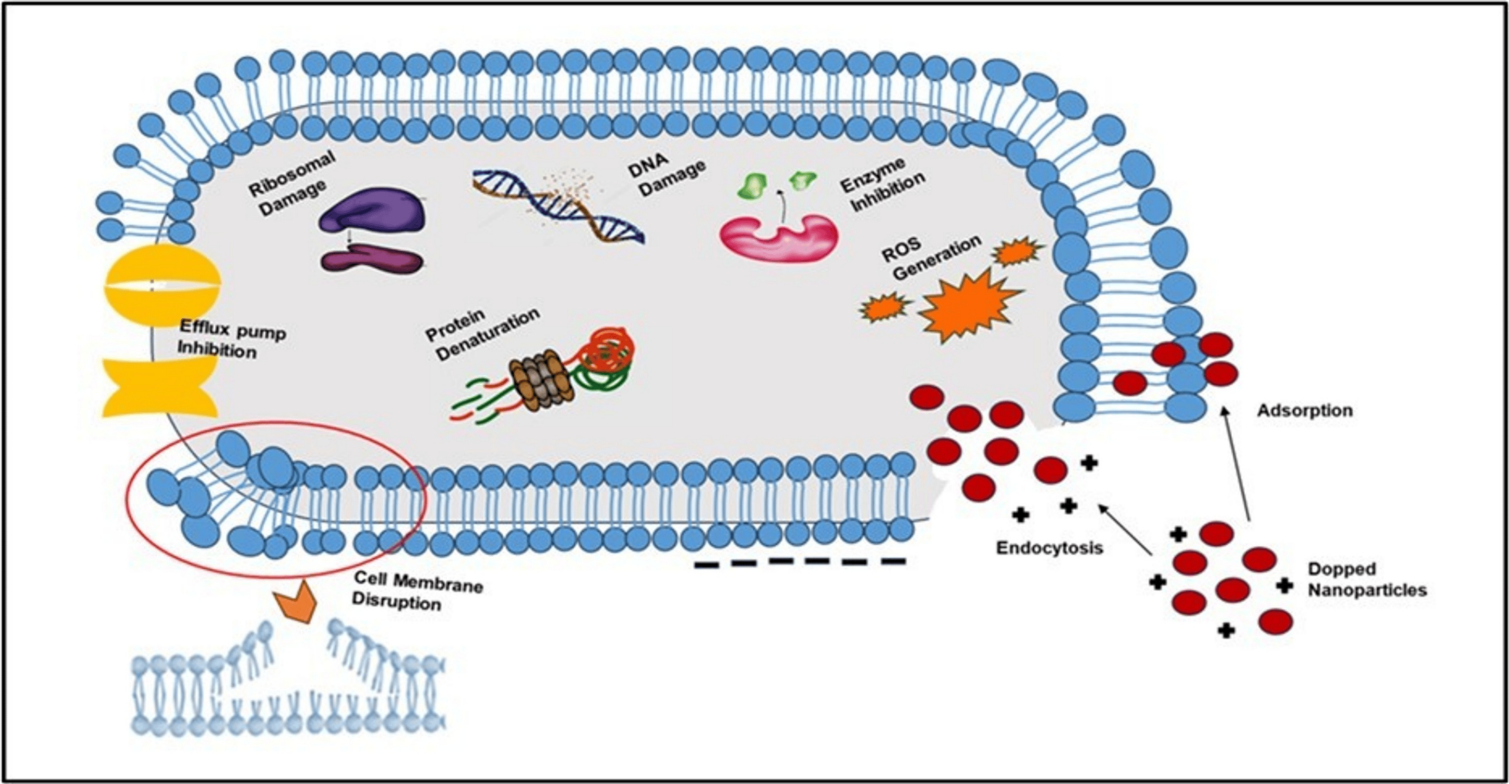
Antimicrobial action of doped IONP's The image was created using Microsoft PowerPoint 3.12 IONP, iron oxide nanoparticle

Doped IONPs seem useful against various conditions, including those spurred on by bacteria, viruses, and fungi. The mechanism behind this antibacterial effect is unclear. A comprehensive investigation has been carried out to understand their mode of operation better, as seen in Figure 3.

Generation of ROS

ROS are created in Fenton reactions and photocatalysis. Being genotoxic, ROS harm DNA molecules. ROS are what produce the oxidative stress [38]. There are several ways in which free radicals can kill bacteria. They can stop enzymes from working, break down carbohydrates, and damage DNA and peroxide membrane lipids [39]. ROS levels increase, and antioxidant system enzymes such as catalase and glutathione reductase may become less active.

Additionally, doped IONPs can affect bacteria's membrane structure [40]. The doping method's ability to combine the foreign metal with nanoparticles can significantly increase ROS generation. Furthermore, doping particles may change the spherical shape of nanoparticles into a structure resembling nanofoils, which increases their antibacterial activity [41].

Inhibition of Antibiotic-Resistant Genes

Doped IONPs can connect to protein functional groups such as mercapto (−SH), carboxyl (−COOH), and amino (−NH). This binding can cause enzymes to stop working or be partially blocked, and it can also lower the production of genes in bacteria that make them resistant to antibiotics [42-43]. Due to the extraordinary qualities that magnetic IONPs possess, they have the potential to transport targeted genes more effectively than other inorganic nanomaterials. These characteristics include a high level of biocompatibility, low toxicity, simple synthesis, and easy chemical functionalization. Additionally, the magnetic responsiveness of the material is exceptional [44-47]. Researchers have recently sought suitable chemical compositions and formulation strategies for mRNA delivery platforms [48]. Two essential benefits suggested by the results were the improvement of r2 reflexivity for magnetic resonance imaging and the increase of mRNA-mediated protein expression. Therefore, they thought that IONPs have great potential for transporting mRNA in the context of theranostic applications [49].

Cell Wall and DNA Disruption

Using the doping technique with transition metals, particularly Cr can affect bacterial DNA replication, transcription, and structure [50]. Like unmodified IONPs, doped IONPs might build up inside the cell and induce harm to its wall. The redox potential is also changed by the F0/F1-ATPase enzyme's function and the speed at which H+ moves across the cell membrane [51]. Because treated IONPs are the same size and shape as other metal IONPs, they have been the subject of many studies to test their antibacterial properties [52].

Efflux Pump Dysfunction

Metal-doped IONPs may turn off the efflux pump in two ways. Doped IONPs may promptly bind to efflux pump active sites to limit antibiotic leakage. The binding of efflux pumps may be impacted by metal-doped nanoparticles functioning as competitive antibiotic inhibitors [51]. A further option is to disrupt efflux kinetics. Metal nanoparticles can change the proton gradient and the proton motive force or membrane potential. This could decrease the force that drives efflux pumps [53-55].

Internalization of Nanoparticles

The vast surface area and high permeability of nanoparticles, particularly smaller nanomaterials, lead them to severely impact the cell walls of microorganisms. The high surface area of IONP helps them adsorb large amounts of substances onto cell membranes, which disrupts transport and the delicate membranes involved. These substances also penetrate the metabolism and have harmful effects by negatively impacting the exchange of matter and energy [56].

Antimicrobial drugs bound to IONPs

The application of magnetic nanoparticles in drug delivery systems is encouraged by their bigger reactive surface and ability to pass biological barriers than their micrometric counterparts. The pharmacological properties of pharmaceuticals may be enhanced by covalent bonding them to IONPs or core-shell nanosystems. This binding may occur via adsorption, polymer matrix dispersion, nucleus encapsulation, electrostatic interactions, or surface covalent attachment [57]. In the following, we shall examine the antifungal and antibiotic properties of IONPs.

Antibiotics

The emergence of very resistant bacteria strains and the absence of conventional antibiotics have generated interest in developing antibiotic-delivery nanosystems using chitosan (CS)-functionalized IONPs as streptomycin carriers [58]. Given in physical combination, this antibiotic demonstrated a rapid release (20 minutes) in phosphate-buffered saline, suggesting that IONPs may function in controlled-release systems. Still, when used as a nanosystem, the whole release process took 350 minutes [59]. Antibiotics such as rifamycin, anthracycline, fluoroquinolone, tetracycline, and cephalosporin were bonded to silver (Ag) nanoparticles that were already in place and contained Ag. It is thought that the positively charged Ag ions interact with the IONPs in a way that makes it possible for the drugs to be joined [60].

In addition, IONPs decorated with Ag were found capable of attaching to rifampicin, doxycycline, cefotaxime, and ceftriaxone. The association mechanisms differed for each antibiotic, involving electrostatic binding with harmful sites and hydrogen bonding. Testing was conducted on synthesizing the Nanosystem IONPs-Ag-rifampicin using the green sonication-assisted procedure [61]. Research on alternative therapeutic resource models has also included the IONPs-ciprofloxacin nanosystem, which has the potential to be enhanced with lactose particles [62]. To create nanosystems, IONPs that are directly coupled to amoxicillin, bacitracin, cefotaxime, gentamicin, kanamycin, neomycin, penicillin, streptomycin, polymyxin, and vancomycin (without shells) have been synthesized [63].

Antifungal Medications

Fungal diseases pose a significant risk to individuals with compromised immune systems. If left untreated, these infections can have serious consequences. Hussein-Al-Ali et al. created a nanosystem comprising IONPs and CS in response to the extensive use of nystatin (NYS) as a fungicide [64]. In contrast to the 1,800-minute release profile of the IONPs-CS-NYS nanosystem, the scientists found that the former persisted for around 20 minutes. Because of the electrostatic interaction between the positively charged CS and the negatively charged NYS, the IONPs-CS-NYS nanosystem created a controlled release of NYS. This phenomenon could be elucidated by the negative charge associated with NYS. This interaction causes difference. Magnetic nanosystems also test ketoconazole and amphotericin B to improve antifungal action and reduce side effects. Immobilization with either bovine serum albumin or human serum albumin is necessary for ketoconazole-functionalized IONP. Because of the hydrophobic contact, the binding mechanism of ketoconazole was successful. Conversely, a mechanism involving amine and aldehyde groups immediately allowed amphotericin B to bind to IONPs. These interactions were noted in the framework of antifungal therapy [65].

Toxicity of IONPs

The field of toxicology focuses on investigating the harmful impacts of various agents, including chemicals, physical factors, and biological substances, on humans, animals, and the environment. Cell toxicity results in decreased mitochondrial function, membrane permeability, and alterations in cell shape. Toxic MNPs, on the other hand, may interfere with the efficacy of the therapy by lowering cell viability, proliferation rate, and metabolic activity [66].

In vitro and in vivo studies of IONP ionophoresis have shown conflicting results. IONPs' toxicity is frequently affected by nanosystem components. Spheric and micrometric IONPs were safer than rod-shaped or nanosized ones [67]. IONP surface charge can potentially influence both the cytotoxicity and genotoxicity of cells. Because they interact with negatively charged cell membranes nonspecifically and via adsorptive endocytosis, positively charged IONPs are more dangerous; increased intracellular accumulation results from this, affecting cell membrane integrity [68].

Understanding the Adverse Impact of IONPs

The production of ROS is believed to contribute to cellular oxidative stress, which may partially clarify which cell lines are most affected by the IONPs' toxicity [69]. IONPs accumulate in the lysosomes, and they decompose into iron ions after being taken up by cells via endocytosis. Perhaps ions might get through membranes and reach vital areas of the cell, such as the mitochondria and nucleus. They then combine with oxygen and hydrogen peroxide to produce ROS [70]. While oxidative stress has been extensively studied as a hypothesis regarding toxicity and cellular damage, it is important to note that excessive iron exposure from IONPs can also lead to severe detrimental effects and cell mortality [71].

Influence of Coatings on the Toxicity of IONPs

IONP surface coating is a commonly used technique to increase the biocompatibility and reduce the toxicity of IONPs. This approach reduces the number of oxidative sites on the nanoparticles, consequently minimizing DNA damage [72]. An in vitro investigation notably demonstrated that uncoated IONPs exerted more pronounced harm on rat fibroblast cells than their polyvinyl alcohol-coated counterparts. This adverse impact was caused by gas vesicles attached to bare particles, changing the proteins' functions and upsetting the ions' balance in cells [73]. Uncoated IONPs also increased intracellular density and promoted ROS generation, both of which may lead to changes in cell morphology. Conversely, protein-coated IONPs achieved densities that induced manageable alterations [74]. The adverse effects of IONPs can also be attenuated by coatings comprising polymers and essential oils. Poly(lactic-co-glycolic acid) (PLGA)-functionalized IONPs affected the endoplasmic reticulum, Golgi body, lysosomes, and mitochondria. This outcome was associated with starting autophagy in the cells [75].

Even though there have been some recorded differences in the research, it is essential to recognize the enormous benefits of IONP protection. Previous studies have shown that D-mannose or poly-l-lysine coatings of IONPs did not lessen their toxicity to mouse neural stem cells. These kinds of nanoparticles disrupt mitochondrial equilibrium [76]. Taking this into consideration highlights the need to perform other tests in addition to cell viability to get a more comprehensive evaluation of the harmful effects of these nanosystems.

IONPs’ potential for improvement and future possibilities

The size, shape, and distribution of IONPs majorly impact their pharmacokinetics and bio-distribution. Nevertheless, numerous methods of nanoparticle preparation encounter a range of limitations. Producing stable, size-controlled, and monodispersed particles with adjustable shapes is formidable. A significant obstacle still lies in producing IONPs that are functionalized, scalable, regulated, repeatable, high-quality, and long-term stable. Further research is required to explore how iron oxides are formed under different conditions [77]. Designing non-agglomerated IONPs with efficient coatings is a significant challenge in all manufacturing techniques. These coatings must deliver optimum performance in biological uses both in vitro and in vivo [78].

Despite significant advances, several obstacles remain in the surface delivery of drug conjugates to IONPs. A primary challenge is the poor entrapment efficiency, as this drug conjugation method is applicable to only a limited range of medications. Additionally, the prolonged covalent bonds between the drug and the particle surface can hinder drug release at the target site [79]. Researchers have addressed this by developing alternative conjugation strategies and employing dynamic bonding approaches to enhance drug release control. The burst release effect, which complicates drug distribution, has also been a significant concern. Efforts to overcome this issue include the use of cross-linkable polymers and advanced coating materials that help mitigate the burst effect and regulate the drug release rate [80]. Furthermore, inadequate surface coatings can lead to premature drug release, posing potential risks. To address this, researchers have explored novel coating materials and techniques to improve the stability and targeting of drug-loaded IONPs. Another challenge lies in the limited understanding of the interactions between IONP surfaces and target tissues, including their adherence and vector presence. Recent studies have focused on optimizing surface modifications and enhancing targeting efficacy to improve therapeutic outcomes. These ongoing efforts highlight the dynamic nature of research aimed at overcoming these challenges and advancing the clinical application of drug-conjugated IONPs.

With a positive outlook, optimizing and developing synthesis technologies further for IONPs to drive progress is essential. The precise adjustment of nanoparticle size is crucial for achieving optimal performance. IONPs need to be continuously improved to keep expanding the limits of biomedical applications. This discovery opens avenues for exploring clinical applications beyond MRI, which is incredibly promising and exciting. There will be an increase in the availability of IONPs-based materials for the consumer market. Due to advances in surface alteration technology and the development of practical, stable, and environmentally acceptable surface alteration chemicals, multifunctional IONPs may soon change nanomedicine and be mass-produced. IONPs have a promising future in multifunctional therapies, early detection, gene delivery, disease diagnostics, and cellular and deep tissue imaging. IONPs will be used in future medical practice through these initiatives, and diagnostic procedures will become quicker, simpler, and less intrusive [81]. When considered collectively, these data suggest that IONPs could potentially bring in a new era for antimicrobial therapy.

## Conclusions

Doped IONPs are shown to have far higher antibacterial activity than their unaltered counterparts. Various factors, such as particle size, coating materials, and fabrication methods, can influence the antibacterial efficacy of doped IONPs. Studies have demonstrated that doped IONPs within a wavelength range of approximately 20 to 200 nm exhibit notable antibacterial effects against various microorganisms. On the other hand, nanoparticles with sizes lower than 20 nm or larger than 200 nm had less antibacterial action. At different calcination temperatures, the dose-dependent inhibition of metal-substituted doped IONPs is caused by their small size and large metal molarities. This phenomenon is attributed to the combined effect of these two conditions. Doping IONPs with metals such as Ag, Au, Zn, and Al has reduced or eliminated toxicity. At the other end of the spectrum, toxic heavy metals and metal oxides, such as Cr and titanium oxide, can potentially aggravate the toxicity of IONPs.

The study of metal-doped materials and their uses, especially in terms of drug transport and antibacterial characteristics, was reviewed in this paper. Promising findings in their capacity to fight both Gram-positive and Gram-negative bacteria indicate that nanoparticles may be helpful in antibacterial agents. It is still unclear and up for debate how doping increases the toxicity of metal oxide nanoparticles against bacteria. No appropriate in vivo models have been available to evaluate doped nanoparticles' important antibacterial characteristics thoroughly. Complete investigation of the antibacterial activity of metal-doped nanoparticles requires extensive in vivo studies. Metal-doped nanoparticles have some prospective hazards, yet their potential advantages should not be underestimated. Exploring the therapeutic potential of metal-doped nanoparticles requires risk assessments using relevant in vivo disease models. How to investigate the problems related to the large-scale production of metal-doped nanoparticles is still unexplored.
